# Activity Theory as a Theoretical Framework for Health Self-Quantification: A Systematic Review of Empirical Studies

**DOI:** 10.2196/jmir.5000

**Published:** 2016-05-27

**Authors:** Manal Almalki, Kathleen Gray, Fernando Martin-Sanchez

**Affiliations:** ^1^ Health and Biomedical Informatics Centre Melbourne Medical School The University of Melbourne Melbourne Australia; ^2^ Faculty of Computer Science and Information Systems Information Systems Department Jazan University Jazan Saudi Arabia; ^3^ Healthcare Policy and Research Weill Cornell Medicine Cornell University New York, NY United States

**Keywords:** activities of daily living, diagnostic self-evaluation, patient activation, patient participation, self-care, self-experimentation, self-management, user-computer interface, activity theory, human-computer interaction, self-quantification, self-tracking, personal informatics, quantified self, self-monitoring

## Abstract

**Background:**

Self-quantification (SQ) is a way of working in which, by using tracking tools, people aim to collect, manage, and reflect on personal health data to gain a better understanding of their own body, health behavior, and interaction with the world around them. However, health SQ lacks a formal framework for describing the self-quantifiers’ activities and their contextual components or constructs to pursue these health related goals. Establishing such framework is important because it is the first step to operationalize health SQ fully. This may in turn help to achieve the aims of health professionals and researchers who seek to make or study changes in the self-quantifiers’ health systematically.

**Objective:**

The aim of this study was to review studies on health SQ in order to answer the following questions: What are the general features of the work and the particular activities that self-quantifiers perform to achieve their health objectives? What constructs of health SQ have been identified in the scientific literature? How have these studies described such constructs? How would it be possible to model these constructs theoretically to characterize the work of health SQ?

**Methods:**

A systematic review of peer-reviewed literature was conducted. A total of 26 empirical studies were included. The content of these studies was thematically analyzed using Activity Theory as an organizing framework.

**Results:**

The literature provided varying descriptions of health SQ as data-driven and objective-oriented work mediated by SQ tools. From the literature, we identified two types of SQ work: work on data (ie, data management activities) and work with data (ie, health management activities). Using Activity Theory, these activities could be characterized into 6 constructs: users, tracking tools, health objectives, division of work, community or group setting, and SQ plan and rules. We could not find a reference to any single study that accounted for all these activities and constructs of health SQ activity.

**Conclusions:**

A Health Self-Quantification Activity Framework is presented, which shows SQ tool use in context, in relation to the goals, plans, and competence of the user. This makes it easier to analyze issues affecting SQ activity, and thereby makes it more feasible to address them. This review makes two significant contributions to research in this field: it explores health SQ work and its constructs thoroughly and it adapts Activity Theory to describe health SQ activity systematically.

## Introduction

People can now measure for themselves their heart rate, sleep quality and quantity, mood, workouts, blood pressure, food consumed, quality of surrounding air—anything from mental, emotional, and physical to social and environmental aspects of daily life—because of advances in wearable sensors and apps, or for short, self-quantification (SQ) tools [1]. Examples of SQ tools are Fitbit for counting steps and tracking sleep, Adidas miCoach for tracking physical activities such as swimming and running, and Lumo Back for monitoring posture. Health SQ has the potential to induce changes in behaviors: according to one US survey [2], 69% of adults tracked themselves; 21% of the study population was using dedicated devices, and 46% stated that they had changed their behavior based on the collected data.

To achieve positive behavior changes, self-quantifiers need to undertake various activities in order to acquire, quantify, and aggregate data about aspects of their personal health, and translate these activities into activities of daily living such as eating healthily, maintaining a healthy weight, and being physically active [3-7]. These activities can be conceptualized as a form of doing work [8]. During this work, users interact with their personal SQ tools in order to collect and reflect on their data [4,5,7,8]. Thus, these tools mediate the SQ work [9-13].

The mediation principle here suggests “a structure for human-computer interaction that (...) the components of the structure should be not only the user and the computer but also the object the user is operating on through the computer application and the other people with whom the user is communicating” [14]. To come to an adequate understanding of this structure of human-computer interaction, deconstruction of the work or overall activity of tool use is needed [15]. This deconstruction can be achieved by breaking down the work into its contextual components or constructs [14-17].

The primary aim of this study was to review studies on health SQ in order to explore the self-quantifiers’ work and activities and to answer the following questions: what constructs of health SQ work have been identified and examined in the relevant literature? How have these studies described such constructs? This review tests our hypothesis that health SQ can be characterized in two main complementary ways: as work on data and work with data.

The secondary aim of this review was to model health SQ constructs, in order to better characterize the SQ work in health. Activity Theory (AT) appears to provide an appropriate conceptual basis to model health SQ work, because the unit of analysis in AT is the activity or work [16,18], within which 6 key constructs are recognized: subject, instruments, objectives, division of work, community, and plans and rules [14-16]. Therefore, this paper tests the adequacy of AT as a way to conceptualize health SQ.

## Methods

### Search Strategy

**Figure 1:**
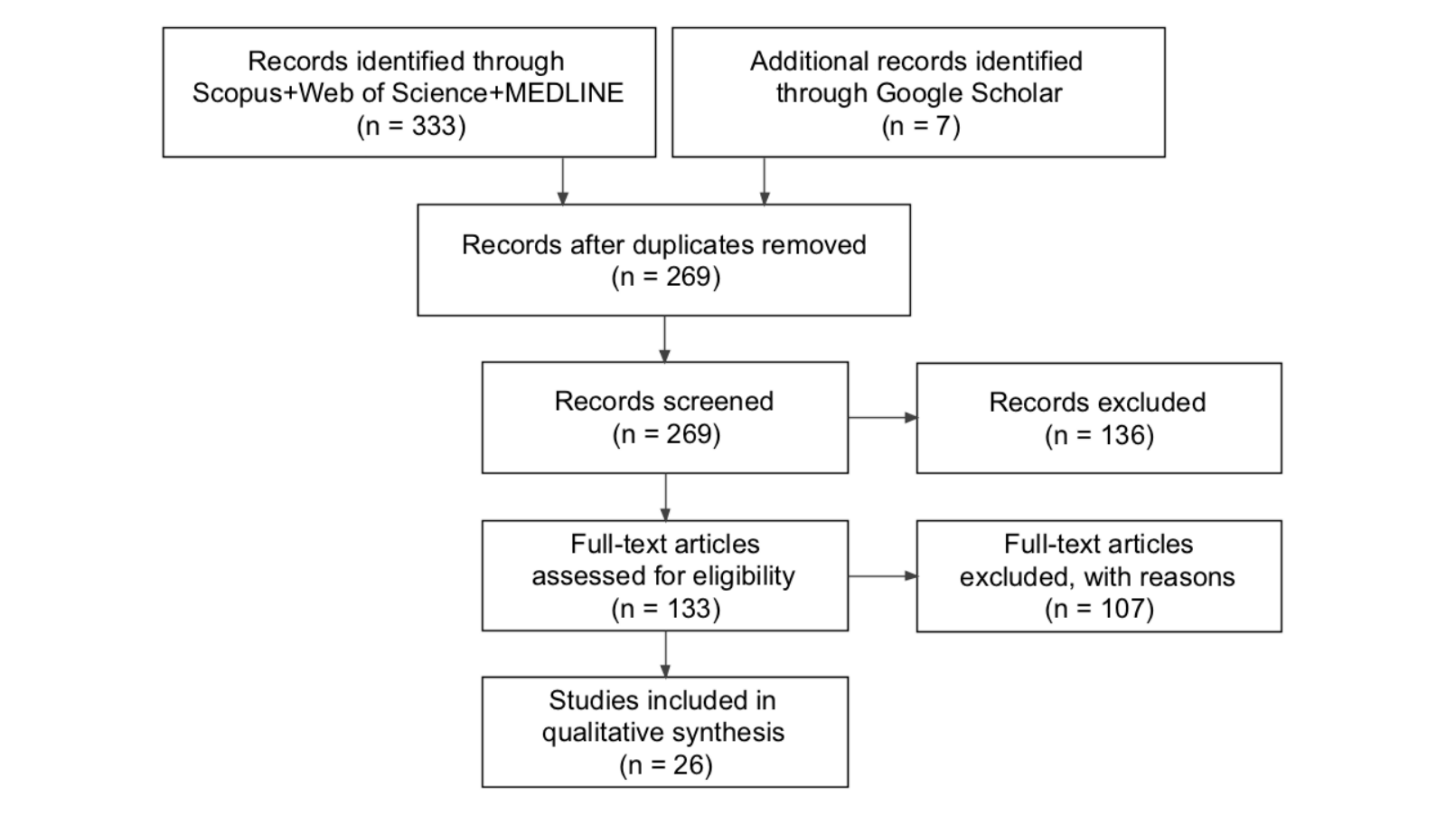
Flow diagram for health self-quantification systematic review.

We searched Scopus, Web of Science, MEDLINE, and Google Scholar. We used the search strings “quantified self,” “quantif*+self*,” and “quantif*AND self.” The search included studies published from January 2007 to October 2015. We included journals, conference proceedings, and papers written in English. The review was guided by the Preferred Reporting Items for Systematic Reviews and Meta-Analyses (PRISMA) statement for reporting systematic reviews [19] (Figure 1).

### Inclusion and Exclusion Criteria

Twenty-six empirical studies met our inclusion criteria (see Multimedia Appendix 1). This sample was achieved through screening titles, abstracts, and keywords of 340 studies (Scopus 211, Web of Science 102, MEDLINE 20, and Google Scholar 7). A total of 71 studies were duplicated; of the remainder, we excluded 243 studies, in two rounds. In the first round, 136 studies were excluded because these studies either did not consider the use of SQ technologies for health self-management or did not investigate users in real-world contexts or daily life settings (rather than in controlled environment laboratories) where people individually collected, managed, and reflected on data. As a result of this round, 133 articles were eligible for the next round of full-text screening. In this round, exclusion criteria were as follows: studies provided conceptual knowledge but not empirical evidence, eliminating 78 articles; and studies focused on proposing a new solution and reporting only its technical specifications, eliminating 29 articles (see Multimedia Appendix 2) [20-122].

### Characteristics and Quality Assessment of Included Studies

To assess the quality of the included studies, we followed the method recommended by Thomas and Harden [123].

Included studies in this review were coded by authors’ names, year of publication, study aims, study design, and study main outcomes or results (Multimedia Appendix 1). Other characteristics noted for each study were as follows: the sampling frame (ie, source of data, selection of participants, recruitment methods, and consent); data collection methods (eg, interviews, questionnaires, and so on); the strategies used to ensure the reliability and validity of data collection methods; data analysis methods (eg, inductive content analysis, statistical quantitative analysis, and so on); and the strategies used to ensure the reliability and validity of data analysis methods.

Included studies in this review were then assessed according to 12 criteria, covering three main quality issues. Five criteria are related to the quality of the reporting of a study's aims and objectives, sampling frame, and data collection and analysis methods (eg, was there an adequate description of the sample used and the methods for how the sample was selected and recruited?). A further 4 criteria are related to the sufficiency of the strategies employed to establish the reliability and validity of data collection and analysis methods, and hence the validity of the findings. The final 3 criteria are related to the appropriateness of the study design (ie, appropriateness of the included study design considering our research aims). The first 9 criteria are suggested by Thomas and Harden [123], whereas the last 3 criteria, related to the appropriateness of the study design, are left open and can be decided by the researcher. In this review, these criteria are assumed to be met by setting the inclusion and exclusion criteria as explained in the previous section, and including only empirical studies for the thematic analysis. We found that all the included studies have reported adequate descriptions of the research aims and objectives, sampling frame, and data collection and analysis methods as well as good or some attempt to establish the reliability and validity of the data collection and analysis methods; thus, they met most of these quality criteria (see Multimedia Appendix 1).

### Extracting Data from Included Studies and Thematic Analysis

To extract and synthesize themes about health SQ work, first we inductively coded the included studies [124,125]. Then, to investigate the constructs of health SQ work in the light of AT, we deductively coded the included studies into the following themes [124,125]. Subject is a person engaged in an activity and using instruments in the course of this work [16]. Object (in the sense of “objective”) is held by the subject and motivates activity, giving it a specific direction [126]. Division of work relates to the extent to which an activity involves collaboration and the sharing of tasks with others [16] or with tools [127]. Community is a group of people with whom the subject shares similar objectives [14,16]. Plans and rules are the norms and specifications of an activity that is undertaken by subjects to fulfill their objectives [16]. Open coding of the content of each study was done by looking at the language it used to explain and describe SQ and associating terms and synonyms with these themes. Both stages of analysis were undertaken by author MA, and then the preliminary results were critiqued jointly by authors KG and FMS, in several rounds of review, until all authors reached agreement on the interpretation of the data.

## Results

### Self-Quantification as Work “On Data” and “With Data”

We found that the literature provided a variety of perspectives on health SQ as data-driven work that users undertake to fulfill their health objectives [4,127,128]. It described how users interact with the SQ tools to define what aspects are relevant to their health conditions (eg, weight, sleep, blood pressure, and so on) [4,5]; set goals and track data about these health aspects for a period of time [7]; analyze the collected data to extract insights on health status [129,130]; adjust behaviors based on the insights and knowledge obtained from the analyzed data [130]; and control the adapted behaviors by sustaining the changes until the desired health outcomes are achieved [127]. We found that it was possible to categorize the overall content of the literature on health SQ activity or work in two ways: work on data and work with data. Table 1 maps these to the included studies. We found that the literature addressed working on data much more that working with data.

The first type, work on data, refers to activities that users carry out to manage their health SQ data. We found that the description of health SQ activities was not consistent across the reviewed literature, even when studies were based on the same human-computer interaction concepts. One study [4] stated that there are five key activities: preparation (eg, determining what information to collect and what collection tool to use), data collection, data integration (ie, combining data from multiple tools into one place), reflection (ie, analyzing data and looking at or exploring visualized information), and action-taking by the tools (eg, sending alerts to the user). However, another study [7] asserted that there are three key activities: goal setting and collecting data via using the tools (eg, BodyMedia—an armband for weight management—allows users to set goals toward more physical activity, lose weight, or stay the same); interpretation or reflection on the data (eg, analyzing data and calculating the time necessary to reach goals by the software); and providing feedback and coaching by the software (eg, Larklife—a wristband that tracks steps taken—will glow in a certain color when the users have not been moving for a while). Furthermore, we found that data collection and analysis activities received high attention, whereas hardly any study examined data handling activity (eg, data organizing, storing, and so on).

The second type, work with data, refers to activities that users carry out in using their data as the basis for actively managing their health status [8,10]. Some studies used the term “reflection” to imply this type of activities; to reflect upon data, users are assumed to look at their analyzed data, attempt to understand them, and act upon this understanding to improve or maintain health status (as in studies [4,7]). Other studies described this kind of interaction from the perspective of users’ cognition in relation to one or more factors: building belief [12,131]; intentions for collecting data [131]; perception of the usefulness of using such tools in tracking health [132]; perception of the usefulness of objective data in making informed decisions [133], building knowledge or awareness about health and function status [5,7,130,134], in deciding what action should be taken on the generated data [8], the ability to act [132], and maintaining behavior or keeping track to cope with growing health problems or conditions [135]; and perception of themselves as good or bad self-quantifiers [8]. However, the definition of these factors is vague and questions remain open about how these factors are interrelated. We found that there is a need for further conceptualization to define and describe this kind of interaction between users and their health SQ data.

### Constructs of Overall Health Self-Quantification Activity

Applying the concepts of AT to our analysis of included studies, we found that one study [8] mentioned all 6 AT constructs, although it did not examine their structure or the relationships among them. The remaining studies mentioned only 3 constructs—user, tools, and objectives. Table 1 maps the AT constructs mentioned in the included studies, and the following paragraphs give detailed examples of how we found these occurring in the literature.

**Table 1:** Mapping of themes to included studies in a systematic review of health self-quantification.

Themes	Article reference number																										%
	4	5	6	7	8	9	10	12	13	127	128	129	130	131	132	133	134	135	136	137	138	139	140	141	142	143
**Inductive themes**																											
																											
**Work on data**																											
	X	X	X	X	X	X	X	X	X	X	X	X	X	X	X	X	X	X	X	X	X	X	X	X	X	X	100
**Work with data**																											
	X	X		X	X		X	X				X	X	X	X	X	X	X									50
**Deductive themes from AT^a^**																											
																											
**Subject or user**																											
	X	X	X	X	X	X	X	X	X	X	X	X	X	X	X	X	X	X	X	X	X	X	X	X	X	X	100
**Instruments or tools**																											
	X	X	X	X	X	X	X	X	X	X	X	X	X	X	X	X	X	X	X	X	X	X	X	X	X	X	100
**Objectives**																											
	X	X	X	X	X	X	X	X	X	X	X	X	X	X	X	X	X	X	X	X	X	X	X	X	X	X	100
**Division of work**																											
	X				X																						8
**Community**																											
	X	X			X	X	X	X	X		X	X	X			X	X	X		X				X	X	X	65
**Plan and rules**																											
			X		X					X																	11.5

^a^AT: Activity Theory.

#### Subject

A subject is a user who “tracks many kinds of data about themselves” [5]. They could be life hackers, data analysts, computer scientists, computer literate, early adopters, health enthusiasts, or productivity gurus [5]. They could also be self-experimenters and use such tools to conduct their own health studies and experiments [5,9,10,136]. They could be people who track themselves diligently and have relatively high technical and mathematical skills [5,8,9,130]. On the other hand, users could be more elderly, less educated, less affluent people, or people with one or more chronic conditions; such people could have more limited skills, less experience of using technology, or less access to SQ tools [8,130,137].

#### Instruments

Instruments are the tools that are used for health SQ. The description of tools in the reviewed studies was loose; tools were variously described as systems, devices, sensors, applications, mobile phone apps, Web-based applications, or software. Such variations might be assumed to be based on the characteristics and features of various technologies; however, this was not always the case. The term “system” at times was used to refer to a Web-based application, such as the prototype described by Li [4], and sometimes was used to refer to the health SQ device and its accompanying software application (such as in studies [138-140]). We note that the term “application” is not self-explanatory; there are many different types of applications such as mobile phone–based applications, Web-based applications, and applications associated with physical sensors (eg, the Fitbit app is associated with the Fitbit clip sensor and cannot be used fully without the sensor). Our analysis of this inconsistency shows a gap in the description of SQ tools and indicates the need for an agreed taxonomy.

#### Objectives

Objectives, in practical terms, are what users aim to achieve (eg, to increase exercise levels) [7]. Setting goals in relation to these objectives is enabled by the tools used [7]. The SQ tools convert these goals into a qualitative form of data (eg, activeness state, such as active or inactive) or a quantitative form of data (eg, number of steps per day) that can be used for illustrating the users’ progress toward accomplishing what they want [4]. These data were described as indicators, health aspects, variables, metrics, parameters, and health factors, based on the researchers’ view of the purpose of SQ work. For instance, the expressions “health indicators” and “health aspects” were used often to describe data obtained through tracking and personal analytics (such as in studies [4-6,12,13,128,135,141]), whereas health “variables,” “metrics,” “parameters,” and “factors” were more associated with data produced as a result of self-experimentation (such as in studies [5,127,136,142]). In addition, these objectives could be described from a health perspective. One study classified these data into five categories based on their health and well-being characteristics: body state (eg, physical and physiological), psychological state and traits, activities (eg, exercise, eating, sleeping), social interactions, and environmental and property states [128]; however, this classification is not adequate to account for all health-related aspects in each category. Our review indicates a need for a classification scheme for describing the SQ data in terms of the health objectives, in a comprehensive and systematic manner.

#### Division of Work

The literature showed that the division of work could be interpreted in two ways. In the first case, the SQ work can be technically divided between the users and the tools used [4]. For example, in data collection, the tools may offer manual or automatic data collection. In manual data collection the user needs to perform the required action (eg, logging food intake manually by using CalorieTracker app), whereas in the automatic mode the tools perform the required action (eg, counting steps automatically by Fitbit) [4].

In the second case, the work is divided between users and others (eg, friends, peers, and so on) [8]. Here, the users not only need to collect data and know numbers, but also need to assess their perception of their own status and achievements to gain a holistic understanding about themselves. To do so, they may share their data or experience with peers [6,13,143]. For example, sharing the exact steps taken in a week with a group of peers allows the person to compare their numbers with others and then accordingly evaluate their own personal activity level [9]. In another example, the users may share their findings from health self-experimentation with others who have run similar experiments in order to compare results and then confirm or disconfirm their own hypotheses [5,136]. In addition, some users go online and share their health concerns; for example, to discuss relevant aspects of their health conditions [143], about which we give further details in the next section.

#### Community

Community refers to the persons with whom the users opt to share their SQ experience. Many SQ tools support sharing data via social sharing features (eg, Fitbit allows users to share their data with groups or other individuals) [134]. Self-quantifiers can voluntarily share their data, results, and so on with others who could be friends, family members, relatives, partners, health care professionals [5,7-9,134,135,141], or peers on online and traditional health support groups (eg, PatientsLikeMe, CureTogether, Quantified Self groups, and so on) [5,137,143] or social networks (eg, Twitter, Facebook, and so on) [134,135,143]. By doing so they hope to get motivated, learn together, aggregate insights, compare results, compete or game, engage in teamwork, and so on. Also, in pursuit of supporting research, self-quantifiers may share their SQ experiences with medical researchers who conduct health-related studies at the population level (eg, crowd-sourcing studies over the Internet like Genomera, DIYgenomics, and so on) [10,137,143] or SQ technology researchers who may ask participants to test a certain technical aspect of a tool in natural settings [4,10,12].

#### Plan and Rules

Plan and rules in AT can refer to the method that users decide on to reach their health objectives. In the examined literature, these plans were described as styles of tracking [6] or data collection plans [127]; approaches of using personal data for health self-management [8]; or improvement plans that users design for improving their lifestyle and adjusting behavior based on the insight obtained from the previous tracking experience [127].

One study [6] suggested five methods: directive toward a goal, such as to either lose or maintain weight; documentary, to keep an eye on things but not to change them; diagnostic, to look for associations between health aspects (eg, tracking medication intake and diet to find out the cause of stomach problems); collecting rewards, to score points or register achievements; and fetishized, that is, driven by the interest in trying out new gadgets and technology. A further four methods were added by another study [8]: to take action (eg, tracking blood glucose to adjust diet or medication); to check progress toward goals but not necessarily for taking actions (eg, tracking cholesterol, and blood count values in anemia); to make sense of the health condition status (eg, checking glucose when users experience symptoms that they suspect indicate hypoglycemia, such as feeling light-headed); and to show logs to health care providers.

On the other hand, the rules of health SQ could be related simply to the regularity and frequency of tracking [4,8]. One study described how some users examine their data periodically for a holistic check on their own health, while others use their data frequently for real-time decisions about their behavior [8]. Another study described how users might collect data several times a day (eg, food consumption), once a day (eg, amount of sleep), several times a week (eg, exercise), or a few times a month (eg, symptoms) depending on their health needs and observations [4].

## Discussion

In health SQ, people must undertake many different activities to transform their objectives into the desired outcome. Preparing, acquiring, organizing, maintaining, retrieving, and reviewing data in order to understand health status are related to data management (DM) [144]; therefore, health SQ can be described as a set of DM activities. However, people must undertake activities that go beyond DM in order to actually take initiative and responsibility for managing their health. This kind of work is related to health management (HM) [145-147]; therefore, health SQ must also be described as a set of HM activities. We could not find any study in the literature that identified all the AT constructs of these activities and examined the relations between them.

This limited current view makes it difficult to holistically investigate the effects of health SQ or systematically determine which “constructs” or components could be key to supporting or undermining users’ abilities to pursue their health objectives, and hence to achieve their desired health outcomes. Thus, a rigorous theoretical framework is needed, one that facilitates deeper, multi-aspect, and more systemic understanding of the SQ activity or interaction between users and tools, highlights all the constructs of such activity, and maps them in a structured way [17,131]. This makes it easier to analyze issues affecting the SQ work or activity, and hence makes it more feasible to address them [17]. The following sections present a way to map the constructs identified throughout the literature, extending AT to develop a *Health Self-Quantification Activity Framework*.

### Mapping All the Constructs of Health SQ Activity: Development of the Health Self-Quantification Activity Framework

**Figure 2:**
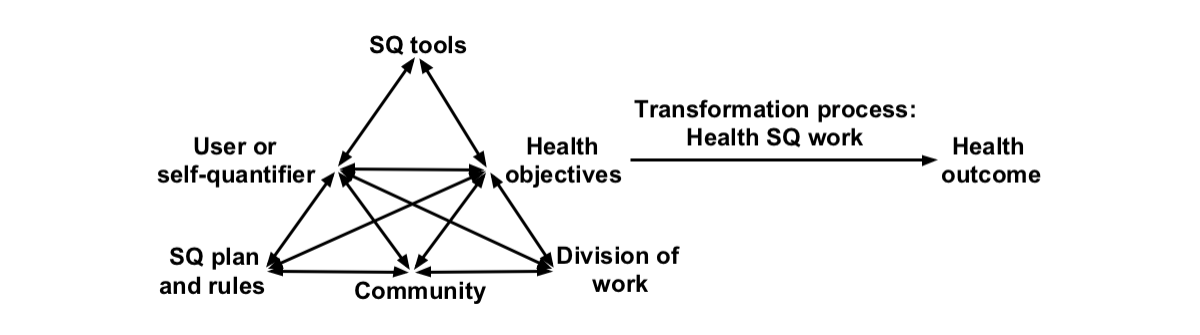
Health Self-Quantification Activity Framework. SQ: self-quantification.

In the AT system, the subject has a goal, tools, colleagues or friends, and rules when he or she is working on the goal to transform it into the intended outcome [15,16,148]. This goal is transformed into outcomes through a process of doing or activities [16]. This process is called the transformation process [16,148]. In this paper, this transformation process represents the work on data and with data that health self-quantifiers perform to manage their own data and health, respectively. In the light of AT, we can say that self-quantifiers have health objectives (eg, being an active or fit person). To attain such objectives, they use SQ tools and set SQ plans to track data in a certain way (eg, directive toward a goal), for example, to walk 10,000 steps per day. They interact with the SQ tools in order to collect, analyze, store, and share data with others as well as to gain knowledge on health, take actions on the generated data to protect or maintain health status, and so on. Thus, AT appears to provide an appropriate conceptual basis to describe health SQ work. Therefore, it is used as a foundation to develop the *Health Self-Quantification Activity Framework* (see Figure 2).

To illustrate how this framework fits with the AT conceptualization of work, the discussion next presents the reconciliation between the AT’s 5 principles and the nature of health SQ practice. These principles constitute the general conceptual system of an activity, which are as follows: object-orientedness, tool mediation, the historical development of activity, the hierarchical structure of activity, and internalization/externalization [149]. Each of these principles will be discussed in turn.

Activity Theory asserts that the subject’s work is an objective-oriented activity [126,150]. Objectives are sense-forming motives [126] where subjects’ consciousness of the world around them is formed by their acting upon it [15]. In addition, an objective can be a motive-stimulus that stimulates a subject to engage in the activity until the desired goals are reached [126]. In health SQ, self-quantifiers track themselves for various purposes or objectives such as to improve health (eg, track blood glucose to hit the target range); to improve other aspects of well-being (eg, track time spent on doing things to be mindful); and to find new life experiences (eg, track heart rate for as long as possible to see what can be learned from it) [5]. Setting goals by using SQ tools in relation to these objectives helps users to collect the data necessary to attain their targets [5,8,13,137]. For example, in case of using Jawbone UP—a wearable wristband for tracking physical activities such as walking—when the users have not been moving a lot, Jawbone UP will start vibrating after being still for a certain time [7]. This helps the users to make sense of their health status (eg, I have not been active today), make a real-time decision (eg, do more walking now), or perform medium-term self-assessment (eg, if a person with diabetes is not feeling well or is inactive today, they may need to check their blood sugar) [8]. It also helps to illustrate one’s progress toward reaching what they want; hence, it stimulates or motivates users to keep on working until their goals are achieved [4-7,13].

Activity Theory asserts that the subjects’ work is mediated by tools [14-16]. Thus, tools could be at the same time both enabling and limiting. They may empower the users during their work to attain their goals, or they may restrict such interaction [15]. Either effect may be observed in health SQ. Self-quantification activities are mediated by the users’ tools [9-13]. As an example of mixed effects, a study about using SQ tools to measure progress by athletes [9] found that if the generated data or scores are below what was expected (ie, because of limitation in data accuracy that users are not aware of), their confidence and how they perceive themselves as athletic individuals may be distorted; consequently, their performance could decrease and eventually they might stop analyzing or even collecting such data.

Activity Theory states that the objective-oriented activity has a history of its own from which the human mind develops an understanding [150]. The historical analysis of the activity is often needed to understand the recent situation [15]. In health SQ, the advances in computational analysis of SQ tools make building a history of work possible, which is a major facilitator to understand current health status and obtain self-knowledge [129,130,134,142]. Data generated from using SQ tools are also beneficial for people to evaluate their future health status. Providing a history of the collected data and detecting trends over time can help users to not only quantify the current health and function status, but also calibrate expectations for upcoming activities based on previous experience. Through evaluating current performance against past performance, they may compare their health status at different times in the year [9,141].

Activity Theory differentiates between activity, actions, and operations to offer a hierarchical structure of activity. Achieving the subjects’ objectives requires them to go through a series of activities [15,16,18]. Each of these activities is composed of actions, and actions are composed of operations [16]. In health SQ, users undertake various DM and HM activities in pursuit of achieving their health objectives. These activities are composed of actions and operations [1]. We can take as an example the data collection activity, which is one of the DM activities: in the case of quantifying the walking habit, it could be composed of several actions such as setting goals, wearing the tools, going for a walk, and so on that are required for generating data or measuring performance [1]. However, the conceptualization of activities and actions in the examined literature is vague; thus, the boundary between them is not clear. This could be one of the reasons for inconsistencies in describing the DM activities, as discussed previously. For example, goal setting was considered a key activity by De Maeyer and Jacobs [7], whereas data collection is an action that constitutes this activity. In contrast, Li [4] considered data collection as a key activity, whereas goal setting is an action that may be a part of this activity.

Activity Theory asserts that any activity has a recursive structure in which feedback that evaluates performance is decisive [150]. On the basis of this feedback, the subject’s internal mental or cognitive actions are formed and this is called internalization. The opposite of the internalization process is the externalization process where internal cognitive actions are transformed into external actions. These two processes demonstrate that cognitive activity is tightly interconnected with external objective-practical activity [150]. In health SQ, data generated from SQ tools may provide decisive feedback about the person’s current health status because they are neutral and not intuitive or emotional [3,8,11,133]. Such data offer an opportunity for users to obtain self-knowledge about health status, and based on this knowledge they decide on what actions they need to take [4,7,9,13]. However, our analysis of the literature shows that these internalization/externalization processes have not been fully investigated yet, and the transition between these two processes remains open for examination. Therefore, a more detailed discussion of these processes cannot be provided in this paper and will be taken up in future work.

### Using the Health Self-Quantification Activity Framework to Investigate Health SQ Work

Many self-quantifiers have been frustrated in accomplishing their health objective [4-10]. They gradually lose their initial motivation and may stop tracking, and thus may not achieve the desired health outcomes [1,4-10,128]. To examine what could cause such a situation in a more systematic and structured way, the *Health Self-Quantification Activity Framework* puts SQ tool use into context, in relation to the goals, plans, and competence of the user. In the following paragraphs, examples of how a person could fail to achieve her or his goals are provided in the light of the health SQ constructs as follows.

“Users” need to have high information and communication technology (ICT) skills [6] and mathematical skills [9] in order to successfully use the SQ tools to track health. However, not everyone is so skilled. A study shows that few people in Europe have high ICT skills (only 29% of the European population from 28 countries) [151]. Therefore, factoring in the users’ technical and mathematical competence levels (eg, low, moderate, or high) could lead to better understanding their health outcomes from health SQ [152,153].

“SQ tools” have a major influence over users. A study found that “users were emotionally involved with their tools, and saw this more as their external conscious to see the details on their physical activity, food and sleep patterns and act on it where necessary” [7]. However, any issues that arise when these tools are in use may increase the time and cognitive effort needed to resolve these issues and to gain self-awareness [4,5,7,154]. For example, if many types of health indicators are of interest, finding a single tool that can support acquisition of most of these data is difficult [4-7,12,138], thus, the users may decide not to collect all the data that would be useful to successfully manage health [4]; if they proceed, they may find themselves using multiple tools, which increases the person’s time and efforts to integrate, analyze, and learn from the collected data [4,5]; and if the same data are tracked by different tools, when combined, data may be inconsistent, giving rise to concerns about data accuracy [139]. Therefore, users may lose their trust in these measurements or may stop tracking [4,10,139,141].

“The division of work”—for example, dividing data collection work between the user and tools in a form of manual or automatic data collection [4,6,10]—may be cumbersome, and this can impede the user’s motivation to collect data of interest or pursue the SQ practice [7]. For example, one study [4] found that self-quantifiers were eager to log their food intake manually at the beginning, but this decreased because as time passed they found it very time consuming.

“Community” is a very real experience that is enabled by health SQ: some users go online and share their data or results about their health status with others who could be friends, family, or strangers, in order to observe, compare, compete, and so on [143]. However, sharing this kind of information has its own set of problems. For example, it could expose the users to privacy breaches and different kinds of discrimination, including discrimination in insurance and other financial dealings [143]. In addition, if the users’ results appeared lower when compared with others, they might consequently develop a low opinion of themselves and thus they might stop tracking their performance [9,137].

“SQ plans and rules” are different for each person, and most SQ systems do not consider this variation. For example, using SQ tools for conducting self-experimentation raises many issues [5,10,136]. When the individuals have personal hypotheses (eg, if I drink sugar water over a period of 8 weeks, then I will lose weight), they want to design their tracking plan to either prove or disprove their hypotheses. However, the lack of integration of single-case experimental design standards into health SQ systems leads to insufficient scientific rigor in data collection and analysis [5,136]. Thus, with current SQ tools, testing one’s thinking about personal weight loss would be challenging and potentially misleading.

### Conclusions

To summarize, health SQ is data-driven and objective-oriented work that is mediated by tools. This work is composed of two types of activities: DM activities (ie, work on data) and HM activities (ie, work with data). These activities comprise 6 constructs (ie, users, SQ tools, health objectives and goals, division of work, community, and SQ plans and rules). Understanding health SQ work, activities, and constructs is important because it is the first step to operationalize health SQ fully. This may in turn help to achieve the aims of health professionals and researchers who seek to make or study changes in the self-quantifiers’ health systematically. This review makes two significant contributions to research in this field: it explores health SQ work and its constructs thoroughly and it adapts AT to describe health SQ activity systematically.

However, many gaps exist in the literature (eg, inconsistency in describing tools, lack of a comprehensive view of health objectives, and vagueness in describing DM and HM activities) that need further investigation. To fill these gaps, our ongoing work is leading toward developing a taxonomy that accounts for various aspects of SQ tools’ functionality and characterizations [1]; a conceptual model and classification schema for explaining data or health aspects [155]; and a model that describes the key DM and HM activities that are necessary for health self-management [1,154]. These are critical contributions to establishing a holistic and rigorous theoretical framework within which to understand and improve health SQ activity.

## Appendix

Multimedia Appendix 1 Included studies, their characteristics, and quality assessment.

Multimedia Appendix 2 Excluded studies and reasons for exclusion.

## Abbreviations

AT: Activity Theory
DM: data management
HM: health management
ICT: information and communication technology
SQ: self-quantification

## References

[1] Almalki M, Gray K, Sanchez F (2015). The use of self-quantification systems for personal health information: big data management activities and prospects. Health Information Science and Systems.

[2] Fox S, Duggan M (2013). Tracking for health.

[3] Lupton D (2014). SSRN: Social Science Research Network.

[4] Li I (2012). Personal informatics and context: using context to reveal factors that affect behavior. Journal of Ambient Intelligence and Smart Environments.

[5] Choe E, Lee N, Lee B, Pratt W, Kientz J (2014). Understanding quantified-selfers' practices in collecting and exploring personal data. CHI '14 Proceedings of the SIGCHI conference on Human factors in computing systems.

[6] Rooksby J, Rost M, Morrison A, Chalmers M (2014). Personal tracking as lived informatics. CHI '14: Proceedings of the SIGCHI Conference on Human Factors in Computing Systems; Toronto, Ontario, Canada.

[7] De Maeyer C, Jacobs A, Matsumura N (2013). Sleeping with technology - designing for personal health. Shikakeology - designing triggers for behavior change: Papers from the 2013 AAAI Spring Symposium.

[8] Ancker JS, Witteman HO, Hafeez B, Provencher T, Wei E, Van de Graaf Mary (2015). “You Get Reminded You're a Sick Person”: Personal Data Tracking and Patients With Multiple Chronic Conditions. J Med Internet Res.

[9] Lee VR, Drake J (2013). Digital Physical Activity Data Collection and Use by Endurance Runners and Distance Cyclists. Tech Know Learn.

[10] FIORE-SILFVAST B, NEFF G (2014). What we talk about when we talk data: Valences and the social performance of multiple metrics in digital health. Ethnographic Praxis in Industry Conference Proceedings.

[11] Saunders W, Krynicki F, Sugarman V (2014). Sisyphorest: maintenance goal support by responding to trends. CHI EA '14 CHI '14 Extended Abstracts on Human Factors in Computing Systems.

[12] Kim J (2014). A qualitative analysis of user experiences with a self-tracker for activity, sleep, and diet. Interact J Med Res.

[13] Gimpe H, Nißen M, Görlitz R (2013). Quantifying the quantified self: A study on the motivations of patients to track their own health. International Conference on Information Systems ICIS 2013.

[14] Kaptelinin V, Nardi B (1995). Activity theory: implications for human-computer interaction. Context and consciousness: activity theory and human-computer interaction.

[15] Kuutti K, Nardi B (1996). Activity theory as a potential framework for human-computer interaction research. Context and consciousness: activity theory and human-computer interaction.

[16] Wilson TD (2008). Activity theory and information seeking. Ann Rev Info Sci Tech.

[17] Nijland N, van LM, Ossebaard HC, Kelders SM, Eysenbach G, Seydel ER, van Gemert-Pijnen Julia E W C (2011). A holistic framework to improve the uptake and impact of eHealth technologies. J Med Internet Res.

[18] Nardi B, Nardi B (1996). Studying context: A comparison of activity theory, situated action models, and distributed cognition. Context and consciousness: Activity theory and human-computer interaction.

[19] Moher D, Liberati A, Tetzlaff J, Altman DG (2009). Preferred reporting items for systematic reviews and meta-analyses: the PRISMA statement. BMJ.

[20] Andrieu B (2016). L’osmose technique avec son corps viv@nt : une auto-santé connectée du patient immersif (Technical osmosis with the living body: Health self-tracking of the immersive patient). L'Évolution Psychiatrique.

[21] Banos O, Amin M, Khan W, Afzel M, Ahmad M, Ali M, Ortuno F, Rojas I (2015). An innovative platform for person-centric health and wellness support. Bioinformatics and biomedical engineering.

[22] Barricelli B, Valtolina S, Diaz P, Pipek V, Ardito C, Jensen C, Aedo I, Boden A (2015). Designing for end-user development in the internet of things. End-User Development.

[23] Chamberlain A, Schraefel M, Poole E, Munson S, Danis C, Churchill E (2015). Moving beyond e-health and the quantified self: the role of CSCW in collaboration, community and practice for technologically-supported proactive health and wellbeing. CSCW'15 Companion: Proceedings of the 18th ACM Conference Companion on Computer Supported Cooperative Work & Social Computing.

[24] Davies N, Friday A, Clinch S, Sas C, Langheinrich M, Ward G, Schmidt A (2015). Security and Privacy Implications of Pervasive Memory Augmentation. IEEE Pervasive Comput.

[25] Doryab A, Frost M, Faurholt-Jepsen M, Kessing LV, Bardram JE (2014). Impact factor analysis: combining prediction with parameter ranking to reveal the impact of behavior on health outcome. Pers Ubiquit Comput.

[26] Dudley JT, Listgarten J, Stegle O, Brenner SE, Parts L (2015). Personalized medicine: from genotypes, molecular phenotypes and the quantified self, towards improved medicine. Pac Symp Biocomput.

[27] Forsdyke DR (2015). Doctor-scientist-patients who barketh not: the quantified self-movement and crowd-sourcing research. J Eval Clin Pract.

[28] Giones-Valls A, Giones F (2015). Quantifying the self to live through data: big data applied to the personal sphere. BiD: textos universitaris de biblioteconomia i documentació.

[29] Hachem S, Mathioudakis G, Pathak A, Issarny V, Bhatia R (2015). Sense2Health a quantified self application for monitoring personal exposure to environmental pollution. Proceedings of 4th International Conference on Sensor Networks, SENSORNETS.

[30] Hood L, Lovejoy JC, Price ND (2015). Integrating big data and actionable health coaching to optimize wellness. BMC Med.

[31] Issa H, Shafaee A, Agne S, Baumann S, Dengel A (2015). Evaluation of Fitbit One, Jawbone Up and Nike+ Fuelband based on Amazon.com customer reviews. Proceedings of the 1st International Conference on Information and Communication Technologies for Ageing Well and e-Health.

[32] Janssen CP, Gould SJ, Li SY, Brumby DP, Cox AL (2015). Integrating knowledge of multitasking and interruptions across different perspectives and research methods. International Journal of Human-Computer Studies.

[33] Jones DH (2015). self-archiving from Christian Boltanski to lifelogging. Archives and Records.

[34] Lavalliere M, Arezes P, Burstein A, Coughlin J (2015). The quantified-self and wearable technologies in the workplace: implications and challenges for their implementations. Sho2015: International Symposium on Occupational Safety and Hygiene.

[35] Lupton D, Jutel A (2015). 'It's like having a physician in your pocket!' A critical analysis of self-diagnosis smartphone apps. Soc Sci Med.

[36] Lupton D (2015). Quantified sex: a critical analysis of sexual and reproductive self-tracking using apps. Cult Health Sex.

[37] Majmudar MD, Colucci LA, Landman AB (2015). The quantified patient of the future: Opportunities and challenges. Healthc (Amst).

[38] Ohlin F, Olsson C (2015). Intelligent computing in personal informatics: Key design considerations. Proceedings of the 20th International Conference on Intelligent User Interfaces.

[39] Olivier B, Vilcocq-Merjagnan C (2015). Online administration of a quantified self-questionnaire for elderly people: a user satisfaction survey. J Am Geriatr Soc.

[40] Picard R, Wolf G (2015). Guest Editorial Sensor Informatics and Quantified Self. IEEE J. Biomed. Health Inform.

[41] Rüping S (2015). [Big data in medicine and healthcare]. Bundesgesundheitsblatt Gesundheitsforschung Gesundheitsschutz.

[42] Thornquist E, Kirkengen AL (2015). The quantified self: closing the gap between general knowledge and particular case?. J Eval Clin Pract.

[43] Tory M, Carpendale S (2015). Personal Visualization and Personal Visual Analytics [Guest editors' introduction]. IEEE Comput. Grap. Appl.

[44] Van den Bulck Jan (2015). Sleep apps and the quantified self: blessing or curse?. J Sleep Res.

[45] Williams SJ, Coveney C, Meadows R (2015). 'M-apping' sleep? Trends and transformations in the digital age. Sociol Health Illn.

[46] Zandi AS, Boudreau P, Boivin DB, Dumont GA (2015). Identification of scalp EEG circadian variation using a novel correlation sum measure. J Neural Eng.

[47] Appelboom G, LoPresti M, Reginster J, Sander CE, Dumont Emmanuel P L (2014). The quantified patient: a patient participatory culture. Curr Med Res Opin.

[48] Becker BW (2014). The Quantified Self: Balancing Privacy and Personal Metrics. Behavioral & Social Sciences Librarian.

[49] Brennan C, McCullagh P, Lightbody G, Galway L, Trainor D (2014). Quantifying brain activity for task engagement. Proceedings of the IEEE International Conference on Bioinformatics and Biomedicine, BIBM.

[50] Bushhousen E (2014). The Quantified Self movement and hospital librarians. Journal of Hospital Librarianship.

[51] Caldwell T (2014). The quantified self: a threat to enterprise security?. Computer Fraud & Security.

[52] Calikli G, Andersen M, Bandara A, Price B, Nuseibeh B (2014). Personal informatics for non-geeks: lessons learned from ordinary people. Proceedings of the 2014 ACM International Joint Conference on Pervasive and Ubiquitous Computing: Adjunct Publication.

[53] Cena F, Likavec S, Rapp A, Deplano M, Marcengo A (2014). Ontologies for quantified self: a semantic approach. Hypertext 2014 extended proceedings of the 25th ACM Hypertext and Social Media conference.

[54] Clifford GD, Gederi E (2014). Out of touch : from audio recordings to phone apps to mattress sensors, noncontact systems offer a less cumbersome way to monitor sleep. IEEE Pulse.

[55] Cuttone A, Larsen J (2014). The long tail issue in large scale deployment of personal informatics. Proceedings of the 2014 ACM International Joint Conference on Pervasive and Ubiquitous Computing: Adjunct Publication.

[56] Cuttone A, Petersen M, Larsen J, Stephanidis C, Antona M (2014). Four data visualization heuristics to facilitate reflection in personal informatics. Universal access in human-computer interaction. Design for all and accessibility practice.

[57] De Croon R, De Buyser T, Klerkx J, Duval E (2014). Applying a user-centered, rapid-prototyping methodology with quantified self: A case study with triathletes. Proceedings of IEEE International Conference on Bioinformatics and Biomedicine (BIBM).

[58] Duarte F, Lourenço A, Abrantes A (2014). Classification of physical activities using a smartphone: Evaluation study using multiple users. Proceedings of the 2013 Conference on Electronics, Telecommunications and Computers.

[59] Gastaldi M (2014). Integration of mobile, big data, sensors, and social media: impact on daily life and business. Proceedings of the 2014 IST-Africa Conference and Exhibition.

[60] Gaunt K, Nacsa L, Penz M (2014). Baby Lucent: pitfalls of applying quantified self to baby products. CHI '14 Extended Abstracts on Human Factors in Computing Systems.

[61] Gurrin C, Smeaton AF, Doherty AR (2014). LifeLogging: Personal Big Data. FNT in Information Retrieval.

[62] Harrison D, Marshall P, Berthouze N, Bird J (2014). Tracking physical activity: problems related to running longitudinal studies with commercial devices. Proceedings of the 2014 ACM International Joint Conference on Pervasive and Ubiquitous Computing: Adjunct Publication.

[63] Hirsch JA, James P, Eastman KM, Conley KD, Evenson KR, Laden F, Robinson Jamaica R M (2014). Using MapMyFitness to Place Physical Activity into Neighborhood Context. Front Public Health.

[64] Hogenboom A, Smit I, Kröse B (2014). Demonstration: a digital coach for self-tracking athletes. Haptics: Neuroscience, Devices, Modeling, and Applications - Proceedings of the 9th International Conference on EuroHaptics.

[65] Huang S, Sano A, Kwan C (2014). The moment: a mobile tool for people with depression or bipolar disorder. Proceedings of the 2014 ACM International Joint Conference on Pervasive and Ubiquitous Computing: Adjunct Publication.

[66] Jain R, Jalali L (2014). Objective Self. IEEE MultiMedia.

[67] Jones C (2014). Tell all. Innovations in Pharmaceutical Technology.

[68] Jordan M, Pfarr N MDDI.

[69] Keary A, Walsh P (2014). How affective computing could complement and advance the quantified self. Proceedings of the Proceedings of the IEEE International Conference on Bioinformatics and Biomedicine, BIBM.

[70] Khorakhun C, Bhatti S (2014). Wellbeing as a proxy for a mHealth study. Proceedings of the IEEE International Conference on Bioinformatics and Biomedicine, BIBM.

[71] Kido T, Swan M (2014). Know Thyself: data driven self-awareness for understanding our unconsciousness behaviors. Big Data Becomes Personal: Knowledge into Meaning: Papers from the AAAI Spring Symposium.

[72] Lagus K (2014). Looking at our data-perspectives from mindfulness apps and quantified self as a daily practice. Proceedings of the IEEE International Conference on Bioinformatics and Biomedicine, BIBM.

[73] Laundav D, Jensen C, Baekgaard P, Petersen M, Larsen J (2014). Your heart might give away your emotions. Proceedings of the IEEE International Conference on Multimedia and Expo Workshops.

[74] Lee V, Briggs M (2014). Lessons learned from an initial effort to bring a quantified self "meetup" experience to a new demographic. Proceedings of the ACM International Joint Conference on Pervasive and Ubiquitous Computing adjunct publication.

[75] Lingg E, Leone G, Spaulding K, B'Far R (2014). Cardea: Cloud based employee health and wellness integrated wellness application with a wearable device and the HCM data store. Proceedings of the 2014 IEEE World Forum on Internet of Things.

[76] Lupton D (2015). Health promotion in the digital era: a critical commentary. Health Promot Int.

[77] Lupton D (2014). Self-tracking modes: Reflexive self-monitoring and data practices. SSRN Journal.

[78] Marcengo A, Buriano L, Geymonat M, Stephanidis C, Antona M (2014). Specch.io: a personal QS mirror for life patterns discovery and “self” reshaping. Universal access in human-computer interaction. Design for all and accessibility practice.

[79] Maturo A (2014). Fatism, self-monitoring and the pursuit of healthiness in the time of technological solutionism. Italian Sociological Review.

[80] Maturo A (2014). M-health and quantified self: developments, potential and risks. Salute e Societa.

[81] Meyer J, Simske S, Siek K, Gurrin C, Hermens H (2014). Beyond quantified self: data for wellbeing. CHI '14 Extended Abstracts on Human Factors in Computing Systems.

[82] Nafus D, Sherman J (2014). Big data, big questions| this one does not go up to 11: the quantified self movement as an alternative big data practice. International journal of communication.

[83] Nikolic-Popovic J, Goubran R (2014). Towards increased usability of noisy ECG signals in HRV-based classifiers. Proceedings of the IEEE International Symposium on Medical Measurements and Applications, MeMeA.

[84] Prince JD (2014). The Quantified Self: operationalizing the quotidien. Journal of Electronic Resources in Medical Libraries.

[85] Rapp A, Cena F, Stephanidis C, Antona M (2014). Self-monitoring and technology: challenges and open issues in personal informatics. Universal access in human-computer interaction. Design for all and accessibility practice.

[86] Rapp A, Hopfgartner F, Plumbaum T, Kay J, Kummerfeld B, Herder E (2014). LinkQS 2014: Linking the Quantified Self Workshop. Hypertext 2014: Extended Proceedings of the 25th ACM Hypertext and Social Media Conference.

[87] Reigeluth T (2014). Why data is not enough: Digital traces as control of self and self-control. Surveillance & Society.

[88] Salamati F, Pasek Z (2014). Personal wellness: Complex and elusive product and distributed self-services. Procedia CIRP.

[89] Schreier G (2014). The internet of things for personalized health. Stud Health Technol Inform.

[90] Shull P, Jirattigalachote W, Hunt M, Cutkosky M, Delp S (2014). Quantified self and human movement: a review on the clinical impact of wearable sensing and feedback for gait analysis and intervention. Gait Posture.

[91] Sjöklint M (2014). The measurable me: the influence of self-quantification on the online user's decision-making process. Proceedings of the 2014 ACM International Symposium on Wearable Computers: Adjunct Program ISWC.

[92] Wenger M, Bell J, McEvoy P, Yamaguchi C, Shokrpour A (2014). Bloom: fostering healthy and peaceful pregnancies with personal analytics. CHI EA '14 Extended Abstracts on Human Factors in Computing Systems.

[93] Zhu Z, Blanke U, Calatroni A, Brdiczka O, Tröster G (2014). Fusing on-body sensing with local and temporal cues for daily activity recognition. Proceedings of the 9th International Conference on Body Area Networks.

[94] Altini M, Penders J, Amft O (2013). Body weight-normalized energy expenditure estimation using combined activity and allometric scaling clustering. Conf Proc IEEE Eng Med Biol Soc.

[95] Buzzo D (2013). Lost time never. Proceedings of the 2013 Inputs-Outputs Conference: An Interdisciplinary Conference on Engagement in HCI and Performance.

[96] Calvo R, Peters D (2013). The irony and re-interpretation of our quantified self. Proceedings of the 25th Australian Computer-Human Interaction Conference: Augmentation, Application, Innovation, Collaboration.

[97] Chen Z, Lin M, Chen F, Lane N, Cardone G, Wang R (2013). Unobtrusive sleep monitoring using smartphones. Proceedings of the 2013 7th International Conference on Pervasive Computing Technologies for Healthcare and Workshops.

[98] Cuttone A, Lehmann S, Larsen J (2013). A mobile personal informatics system with interactive visualizations of mobility and social interactions. Proceedings of the 1st ACM international workshop on Personal data meets distributed multimedia.

[99] Derksen G, Ruecker S, Causer T, Terras M (2013). Demonstrating data using storyboard visualization tool. Proceedings of the 6th International Symposium on Visual Information Communication and Interaction.

[100] Jain R (2013). What's in it for me? how can big multimedia aid quantified-self applications. Proceedings of the 1st ACM international workshop on Personal data meets distributed multimedia.

[101] Kido T, Swan M (2013). Exploring the mind with the aid of personal genome: citizen science genetics to promote positive well-being. http://www.aaai.org/ocs/index.php/SSS/SSS13/paper/view/5736.

[102] Kunze K, Iwamura M, Kise K, Uchida S, Omachi S (2013). Activity recognition for the mind: Toward a cognitive "Quantified Self". Computer.

[103] Lupton D (2013). Quantifying the body: monitoring and measuring health in the age of mHealth technologies. Critical Public Health.

[104] Lupton D (2013). Understanding the Human Machine [Commentary]. IEEE Technol. Soc. Mag.

[105] Martin-Sanchez F, Lopez-Campos G, Gray K, Sarkar IN (2014). Biomedical informatics methods for personalized medicine and participatory health. Methods in biomedical informatics: a pragmatic approach.

[106] Matassa A, Rapp A, Simeoni R (2013). Wearable accessories for cycling: tracking memories in urban spaces. UbiComp Adjunct '13 Proceedings of the 2013 ACM Conference on Pervasive and Ubiquitous Computing adjunct publication.

[107] Mcfedries P (2013). Tracking the quantified self [Technically speaking]. IEEE Spectr.

[108] Spohrer JC, Freund LE, Pasek Z, Bacioiu G (2012). Self-service for personal health monitoring and decisions. Advances in the Human Side of Service Engineering.

[109] Salamati F, Pasek Z (2013). Modeling for personal well-being: time for paradigm change. Proceedings of the 2013 Grand Challenges on Modeling and Simulation Conference.

[110] Swan M (2013). Next-generation personal genomic studies: extending social intelligence genomics to cognitive performance genomics in quantified creativity and thinking fast and slow.

[111] Yumak Z, Pu P (2013). Survey of Sensor-Based Personal Wellness Management Systems. BioNanoSci.

[112] Bottles K (2012). Will the quantified self movement take off in health care?. Physician Exec.

[113] Lathia N (2012). Using idle moments to record your health via mobile applications. Proceedings of the 1st ACM workshop on Mobile systems for computational social science.

[114] Lathia N (2012). Using ratings to profile your health. Proceedings of the 6th ACM conference on Recommender systems.

[115] Li I, Medynskiy Y, Froehlich J, Larsen J (2012). Personal informatics in practice: improving quality of life through data. CHI '12 Extended Abstracts on Human Factors in Computing Systems.

[116] Smarr L (2012). Quantifying your body: a how-to guide from a systems biology perspective. Biotechnol J.

[117] Rivera-Pelayo V, Zacharias V, Mller L, Braun S (2012). Applying quantified self approaches to support reflective learning. Proceedings of the 2nd International Conference on Learning Analytics and Knowledge.

[118] Swan M (2012). Crowdsourced health research studies: an important emerging complement to clinical trials in the public health research ecosystem. J Med Internet Res.

[119] Swan M (2012). Health 2050: the realization of personalized medicine through crowdsourcing, the quantified self, and the participatory biocitizen. Journal of Personalized Medicine.

[120] Swan M (2012). Sensor Mania! The Internet of Things, Wearable Computing, Objective Metrics, and the Quantified Self 2.0. JSAN.

[121] (2012). The Economist.

[122] Swan M (2009). Emerging patient-driven health care models: an examination of health social networks, consumer personalized medicine and quantified self-tracking. Int J Environ Res Public Health.

[123] Thomas J, Harden A (2008). Methods for the thematic synthesis of qualitative research in systematic reviews. BMC Med Res Methodol.

[124] Hsieh H, Shannon SE (2005). Three approaches to qualitative content analysis. Qual Health Res.

[125] Elo S, Kaariainen M, Kanste O, Polkki T, Utriainen K, Kyngas H (2014). Qualitative Content Analysis: A Focus on Trustworthiness. SAGE Open.

[126] Kaptelinin V (2005). The Object of activity: Making sense of the sense-maker. Mind, Culture, and Activity.

[127] Liang Z, Martell M (2015). Framing self-quantification for individual-level preventive health care. Proceedings of the 8th International Conference on Health Informatics HEALTHINF 2015.

[128] Oh J, Lee U (2015). Exploring UX issues in quantified self technologies. Eighth International Conference on Mobile Computing and Ubiquitous Networking (ICMU).

[129] Whooley M, Ploderer B, Gray K (2014). On the integration of self-tracking data amongst quantified self members. Proceedings of the 28th International BCS Human Computer Interaction Conference HCI 2014; Southport, UK.

[130] Choe EK, Lee B, Schraefel MC (2015). Characterizing Visualization Insights from Quantified-Selfers' Personal Data Presentations. IEEE Comput Graph Appl.

[131] Kim J (2014). Analysis of health consumers' behavior using self-tracker for activity, sleep, and diet. Telemed J E Health.

[132] Doyle J, Walsh L, Sassu A, McDonagh T (2014). Designing a wellness self-management tool for older adults: results from a field trial of YourWellness. PervasiveHealth '14 Proceedings of the 8th International Conference on Pervasive Computing Technologies for Healthcare.

[133] Papi E, Belsi A, McGregor AH (2015). A knee monitoring device and the preferences of patients living with osteoarthritis: a qualitative study. BMJ Open.

[134] Shih P, Han K, Poole E, Rosson M, Carroll J (2015). iConference 2015 Proceedings.

[135] Ivanov A, Sharman R, Rao HR (2015). Exploring factors impacting sharing health-tracking records. Health Policy and Technology.

[136] Chang J (2012). Self-tracking for distinguishing evidence-based protocols in optimizing human performance and treating chronic illness. Self tracking and collective intelligence for personal wellness: Papers from the 2012 AAAI Spring Symposium.

[137] Lee V (2014). What's happening in the "Quantified Self" movement?. Learning and becoming in practice: proceedings of International Conference of the Learning Sciences, ICLS 2014.

[138] Packer H, Buzogany G, Smith D, Dragan L, Kleek M, Shadbolt N (2014). The editable self: a workbench for personal activity data. CHI EA '14 Extended Abstracts on Human Factors in Computing Systems.

[139] Guo F, Li Y, Kankanhalli M, Brown M (2013). An evaluation of wearable activity monitoring devices. Proceedings of the 1st ACM international workshop on Personal data meets distributed multimedia; Barcelona, Spain.

[140] Punnoose B, Gray K (2015). Comparative evaluation of two systems for integrating biometric data from self-quantification. Health Information Science. Lecture Notes in Computer Science.

[141] Lee V, Drake J (2013). Quantified recess: design of an activity for elementary students involving analyses of their own movement data. IDC '13: Proceedings of the 12th International Conference on Interaction Design and Children.

[142] Dontje ML, de GM, Lengton RR, Krijnen WP, van der Schans Cees P (2015). Measuring steps with the Fitbit activity tracker: an inter-device reliability study. J Med Eng Technol.

[143] Pickard K, Swan M (2014). Big desire to share big health data: A shift in consumer attitudes toward personal health information. Big data becomes personal: Knowledge into meaning - 2014 AAAI Spring Symposium Series.

[144] Jones W (2008). Personal Information Management. Ann Rev Info Sci Tech.

[145] Hibbard JH, Mahoney ER, Stock R, Tusler M (2007). Do increases in patient activation result in improved self-management behaviors?. Health Serv Res.

[146] Hibbard JH, Peters E, Dixon A, Tusler M (2007). Consumer competencies and the use of comparative quality information: it isn't just about literacy. Med Care Res Rev.

[147] Fowles JB, Terry P, Xi M, Hibbard J, Bloom CT, Harvey L (2009). Measuring self-management of patients' and employees' health: further validation of the Patient Activation Measure (PAM) based on its relation to employee characteristics. Patient Educ Couns.

[148] Mursu A, Luukkonen I, Toivanen M, Korpela M (2006). Activity theory in information systems research and practice: theoretical underpinnings for an information systems development model. Information Research.

[149] Kaptelinin V, Nardi B (1997). Activity theory: basic concepts and applications. CHI EA '97 Extended Abstracts on Human Factors in Computing Systems.

[150] Bedny G, Karwowski W (2004). Activity theory as a basis for the study of work. Ergonomics.

[151] (2014). Eurostat.

[152] Kayser L, Kushniruk A, Osborne RH, Norgaard O, Turner P (2015). Enhancing the effectiveness of consumer-focused health information technology systems through eHealth literacy: a framework for understanding users' needs. JMIR Human Factors.

[153] Sherer S (2014). Patients are not simply health it users or consumers: the case for “e Healthicant” applications. Communications of the Association for Information Systems.

[154] Almalki M, Sanchez F, Gray K (2015). Quantifying the Activities of Self-quantifiers: Management of Data, Time and Health. Stud Health Technol Inform.

[155] Almalki M, Gray K, Martin-Sanchez F (2014). Classification of data and activities in self-quantification systems. http://ceur-ws.org/Vol-1149/bd2014_almalki.pdf.

