# Supplementary material for: Activity Theory as a Theoretical Framework for Health Self-Quantification: A Systematic Review of Empirical Studies
Source: J Med Internet Res. 2016 May 27;18(5):e131. doi: 10.2196/jmir.5000 (PMC4909388; doi:10.2196/jmir.5000)
Supplement: Multimedia Appendix 1 [file jmir_v18i5e131_app1.pdf]

First author (alphabetical order)	Study aims	Study design	Sampling, selection, recruitment, and consent	Data collection methods	Reliability and validity of data collection methods	Data analysis methods	Reliability and validity of data analysis methods	Study main outcomes	Quality criteria met*
2015									
Ancker [8]	Explore the individuals tracking of their own health and medical data.	Qualitative exploratory method used to collect data about personal tracking.	*Sampling source:* patients with multiple chronic diseases, and health care providers. *Selection*: patients from Weill Cornell Medical College and the Institute for Family Health. *Recruitment*: via outpatient clinics in internal medicine and endocrinology and from the patient information library, using both promotional flyers and individual referrals from physicians and nurse practitioners. *Consent***:** written and oral consent were obtained from participants.	Semi-structured interview study with 22 patients and 7 health care providers.	*Reliability*: semi-structured interviews were used to improve the reliability of the collected data. *Validity*: one researcher attended six 90-minute sessions of a diabetes education support group as a means of triangulating emerging themes.	The interviews were transcribed and themes inductively analysed.	*Reliability*: codebook was developed by two researchers, and was reviewed by at least two team members, independently. They then met to reach consensus on it to ensure reliability. *Validity*: researchers used member-checking method to improve internal validity.	Found that (1) tracking such data feels like work for many patients, (2) personal medical data for individuals with chronic conditions are not simply objective facts, but instead provoke strong positive and negative emotions, value judgments, and diverse interpretations, (3) patients track for different purposes, ranging from sense-making to self-management to reporting to the doctor, and (4) patients often notice that physicians trust technologically measured data such as lab reports over patients' self-tracked data.	A, B, C, D, E, F, G, H, I, J, K, L
Choe [130]	Examine what insights people gain from their personal data, and how they use visualisations to communicate their insights.	Qualitative research was conducted by collecting data from video-recordings posted on Quantifies-self Meetup site.	*Sampling source:* members of Quantified Self Meetup group. *Selection*: video-recordings posted voluntarily by the members. *Recruitment*: NA* *Consent***:** NA	30 video-recordings of QuantifiedSelf Meetup talks.	*Reliability*: systematic capturing of the demographic data and information related to key aspects in the study. *Validity*: setting criteria of inclusion.	The video-recordings were transcribed, and themes inductively analysed.	*Reliability*: two researchers coded data independently. They then met to resolve any disagreements or modify the initial coding scheme. *Validity*: the content were analysed repeatedly until data saturation was reached.	Found that there are eight insight types (detail, self-reflection, trend, comparison, correlation, data summary, distribution, and outlier).	A, B, C, D, E, F, G, H, I, J, K, L
Dontje [142]	Determine the inter-device reliability of activity trackers in measuring steps.	Idiographic study was conducted by using Fitbit Ultra activity tracker.	*Sampling source:* the researcher is the subject of study. *Selection*: NA *Recruitment*: NA *Consent***:** NA	The researcher collected data about himself routinely for a period of 8 days, by using ten Fitbit Ultra devices.	*Reliability*: consistent log of steps taken was carried out. *Validity*: one person collected data through the use of multiple tools to ensure the consistency of collecting data method.	Quantitative statistical analysis was conducted to analyse the collected data.	*Reliability*: statistical analysis was performed by a statistical program. *Validity*: evaluation of agreement of producing similar numbers by one device was	Found that individuals can reliably compare their daily physical activity scores with peers.	A, B, C, D, E, F, G, H, I, J, K, L

							based on comparing measures generated from this device with other 9 devices.		
Ivanov [135]	Examine the sharing of health-tracking records (HTR) by patients with others (e.g., spouse/partner, relatives, doctors, etc.).	Exploratory study was conducted by interviewing people over telephone, including landline and cellphone.	*Sampling source:* 3014 adults living in the United States. *Selection*: stratified sampling used to recruit participants who have access to either a landline or a cellphone; telephone numbers were drawn with equal probabilities. *Recruitment*: made as many as 7 attempts to contact every sampled telephone number. *Consent***:** not stated.	Structured interviews with 761 adults who met the inclusion criteria (i.e., had shared their HTR either online or offline, and kept track of their own health related indicators).	*Reliability*: data were systematically collected via interviews. *Validity*: the sample size was big enough for data analysis, and setting criteria of inclusion increased the validity of data collection.	Quantitative analysis to investigate the influence of motivation, health severity, age, and perceived health status factors on sharing of HTR with others.	*Reliability*: use of statistical software for data analysis. *Validity*: multinomial logistic regression was conducted to find significant results.	Results indicate that motivation, health severity, and age substantially increase the likelihood of sharing HTR with doctors in comparison to other categories. In contrast, perceived health status increases the likelihood of sharing data with others as opposed to with doctors.	A, B, C, D, E, F, G, H, I, J, K, L
Liang [127]	Explore preventive health care at the individual level based on self-quantification.	Case study.	*Sampling source:* one healthy adult. *Selection*: a 25-year-old healthy Asian female with no medical history of any significant sickness or chronic diseases. *Recruitment*: not stated. *Consent***:** not stated.	The subject designed and conducted a self health care project for tracking sleep quality for 20 days.	*Reliability*: a diary was used to collect data where the subject kept logging all the potential affecting factors on a daily basis. All data were stored in an Excel file. *Validity*: valid metrics (e.g., Wake-Up Freshness WUF and Subjective Sleep Efficiency SSE) were used to collect data about sleep quality, and a literature review was performed to define the potential affecting factors.	Quantitative analysis of the correlation between each potential affecting factor and the metrics was conducted.	*Reliability*: a reliable statistical method, the Pearson correlation coefficient, was used to analyse data. *Validity*: the result of the quantitative analysis that was used to find the significant factors was clearly reported in two tables.	The result was used to develop a Preventive Health care on Individual Level (PHIL) framework that guides people to leverage their self-tracking data to improve personal health.	A, B, C, D, E, F, G, H, I, J, K, L
Oh [128]	Explore user experience (UX) issues related to quantified self tools, and categorise tracking tools.	Qualitative research was conducted by collecting data from tools reviews posted on Quantifies Self website.	*Sampling source:* members of Quantified Self Meetup group. *Selection*: tools' reviews posted by users on the Quantified Self website. *Recruitment*: NA *Consent***:** NA	209 reviews from the Quantified Self website were collected.	*Reliability*: systematic capturing of the demographic data and information related to key aspects of the study. *Validity*: setting criteria of	An inductive content analysis was conducted to generate themes.	*Reliability*: use of Affinity method to systematically analyse the postings. *Validity*: the extraction of themes was performed iteratively.	Found various tracking categories (e.g., body state, activities, etc.). Also, found many UX issues associated with quantified self tools (e.g., data controllability, integration, accuracy, etc.)	A, B, C, D, E, F, G, H, I, J, K, L

					inclusion.				
Papi [133]	Identify perspective of patients with osteoarthritis (OA), in particular design requirements and mode of use, on wearable technology to support rehabilitation.	Qualitative study using a focus group approach.	*Sampling source:* patients with osteoarthritis.<br>*Selection*: participants were sampled based on being diagnosed with OA through clinical assessment or imaging, undergoing rehabilitation, and having a good understanding of written and spoken English.<br>*Recruitment*: patients were recruited from the Imperial College NHS Trust physiotherapy departments and local communities via poster advertisements.<br>*Consent*: all participants provided written informed consent prior to taking part in the study.	21 patients with OA took part in one of four focus groups.	*Reliability*: a semi structured topic guide was used to collect data.<br>*Validity*: setting criteria of inclusion increased the validity of data collection.	Each focus group was audio-recorded and transcribed verbatim to allow subsequent analysis.	*Reliability*: a thematic analysis was conducted on each focus group by using Framework Methodology.<br>*Validity*: the extraction of themes was conducted iteratively, until data saturation was reached.	Found that the main determinants of user acceptance of a wearable technology were appearance and comfort during use.	A, B, C, D, E, F, G, H, I, J, K, L
Punnoose [140]	Examining TicTrac and HealthVault tools' performance in terms of usability and visualisation in integrating biometric data from self-quantification.	Idiographic study was conducted by using four tools to collect data (LARK sleep monitoring system; Fitbit Atria; Polar wristband; iHealth Blood Pressure), and two tools to integrate data (TicTrac and HealthVault).	*Sampling source:* the researcher was the subject of study.<br>*Selection*: NA<br>*Recruitment*: NA<br>*Consent***:** NA	The researcher collected and integrated biometric data about himself routinely for a period of four weeks, four using SQ tools.	*Reliability*: consistent log of health indicators like sleep, blood pressure, etc., and of measures and time taken to collect and integrate data<br>*Validity*: one person collected data through the use of multiple tools to ensure the consistency of collecting data method.	Data analysis was done by thematically reviewing the content of the log, and by inspecting the resulting visual artefacts that were created using each integrating tool.	*Reliability*: analysis of integration tools performance was done by using models like cognitive efficiency model of graphical design, and usability models.<br>*Validity*: evaluation was based on the measures of usability and visualisation that were identified from the literature.	Found that Tictrac peformed better than HealthVault in both evaluations.	A, B, C, D, E, F, G, H, I, J, K, L
Shih [134]	Explore how personal preferences and other individual characteristics affect use and adoption of wearable activity trackers.	User trial.	*Sampling source:* undergraduate students.<br>*Selection*: undergraduate students who were at a large American university; ranging in age from 20 to 24 years old; and interested in trying Fitbit for tracking self.<br>*Recruitment*: the authors advertised the study in the university to recruit the participants.<br>*Consent***:** not stated.	A six-week user trail was conducted with 18 males and 8 females. Each participant received Fitbit Force and Fitbit Ultra.	*Reliability*: a pre-survey and post-survey that included 7-point Likert scale questions and open questions was used to collect data.<br>*Validity*: setting criteria of inclusion increase the validity of data collection.	Quantitative and qualitative analysis was conducted to analyse the collected data.	*Reliability*: a reliable statistical method, the k-mean, was used to analyse numerical data. Also, thematic coding was used to analyse responses to the open questions.<br>*Validity*: in the quantitative analysis,	Identified a list of challenges that have impact on the use and adaption of wearable activity trackers such as issues with physical design and aesthetics.	A, B, C, D, E, F, G, H, I, J, K, L

							significant results were reported. In the qualitative one, results were coded iteratively by two researchers.		
2014									
Choe [5]	Investigate how users used self-tracking technologies and built workarounds to overcome barriers.	Qualitative research was conducted by collecting data from video-recordings posted on Quantifies Self Meetup site.	*Sampling source:* members of Quantified Self Meetup groups.<br>*Selection*: video- recordings posted by members.<br>*Recruitment*: NA<br>*Consent***:** NA	52 video-recordings of QuantifiedSelf Meetup talks.	*Reliability*: systematic capturing of the demographic data and information related to key aspects in the study.<br>*Validity*: setting criteria of inclusion.	The video-recordings were transcribed, and themes inductively analysed.	*Reliability*: use of Affinity method to systematically analyse the video transcript.<br>*Validity*: the extraction of themes was performed iteratively.	Found that key barriers of self-tracking were tracking too many things, which led to 'tracking fatigue'; not tracking context, which led to not gaining insights; and insufficient scientific rigor.	A, B, C, D, E, F, G, H, I, J, K, L
Doyle [132]	Examine how best to support older adults in health self-management practice	Field trial by using YourWellness – an application that supports elderly in self-reporting on their wellbeing.	*Sampling source:* older adults.<br>*Selection*: people over the age of 60, and those who could provide informed consent.<br>*Recruitment*: not stated.<br>*Consent***:** sought from participants before the trial commenced.	A 5-month home deployment of YourWellness with 8 older adults.	*Reliability*: systematic extraction of data through the use of YourWellness application, and other protocols like think-aloud.<br>*Validity*: the application's validity was tested as a wellness tool in workshops with clinicians, before the study commenced.	Interviews were transcribed, and their content was analysed qualitatively and quantitatively.	*Reliability*: data were analysed by using multiple tools such as System Usability Questionnaire, and Intrinsic Motivation Inventory (IMI).<br>*Validity*: quantitative analysis used to test the significance of the extracted themes.	Found that older adults are interested in self-managing their wellness; they want to be more aware of their health. Presented Other results around how best to design wellness self-management applications for this population.	A, B, C, D, E, F, G, H, I, J, K, L
Kim [12]	Investigate user experiences of using self-tracker tools for activity, sleep, and diet.	Qualitative and quasi-experimental research methods were used to conduct the study.	*Sampling source:* female university students who have no chronic diseases.<br>*Selection*: snowball sampling was used to recruit students.<br>*Recruitment*: the authors approached and verbally recruited the participants.<br>*Consent***:** sought from participants during the orientation session.	Three months study of actual Fitbit utilisation with 44 female college students; and a follow-up in-depth interview with 18 students.	*Reliability*: systematic extraction of constructs from the experimental data was performed.<br>*Validity*: the extraction of constructs was conducted iteratively, until data saturation was reached.	The interviews were transcribed and thematically analysed.	*Reliability*: structured qualitative analysis of literature was conducted.<br>*Validity*: use of quasi-experimental research to validate the results of the literature review.	Developed and verified the Health Information Technology Acceptance Model-II (HITAM-II) that consists of 5 factors: information technology factors, personal factors, social factors, attitude, behavioural intention, and behaviour.	A, B, C, D, E, F, G, H, I, J, K, L

Kim [131]	Verify the health information technology acceptance model (HITAM) in the context of the health consumers' attitude, behavioural intention, and behaviour regarding utilising self-trackers.	Quantitative research method was conducted by using questionnaire survey.	*Sampling source:* female university students who have no chronic diseases.<br>*Selection*: snowball sampling was used to recruit participants.<br>*Recruitment*: the authors approached and verbally recruited the participants.<br>*Consent***:** sought from participants during the orientation session.	44 female college students were asked to register and create an account on the product web site and to wear Fitbit for 90 days to track sleep, diet, and activity.	*Reliability*: systematic collection of data was conducted.<br>Validity: triangulated qualitative methods (e.g., interviews, questionnaires, etc.) were used to collect data and improve the validity of the study.	Statistical analysis of constructs and related variables was conducted.	*Reliability*: descriptive statistics of the variable scores, and the reliability coefficients of the constructs were reported.<br>*Validity*: the validity of the extracted constructs was assessed using statistical analysis, and only statistically significant variables were only included in the HITAM model.	Verified 5 factors in the HITAM model: information technology factors, personal factors, social factors, attitude, behavioural intention, and behaviour.	A, B, C, D, E, F, G, H, I, J, K, L
Lee [137]	Identify the variety of self-quantifiers, the reported motivations driving individuals to self-quantify, and the data analysis activities.	Qualitative research was conducted by collecting data from video-recordings posted on Quantified Self Meetup site.	*Sampling source:* members of Quantified Self Meetup groups.<br>*Selection*: video- recordings posted voluntarily by self-quantifiers.<br>*Recruitment*: NA<br>*Consent***:** NA	12 video-recordings of QuantifiedSelf Meetup talks.	*Reliability*: systematic capturing of the demographic data and information related to key aspects in the study.<br>*Validity*: limitations and biases associated with the use of these videos as source of data were reported.	The video-recordings were transcribed, and themes analysed.	*Reliability*: thick description of the coding process.<br>*Validity*: the video transcripts were coded iteratively.	Provided insights about motivations for pursuing self-quantification, and about enabling conditions that could build on initial motivation and support the presenter's ability to pursue self-quantification and analyses of self-quantification data.	A, B, C, D, E, F, G, H, I, J, K, L
Packer [138]	Investigate users' perceptions of problems with using tracking tools and interpreting data.	Cross-sectional survey collecting qualitative data about self-tracking.	*Sampling source:* not stated.<br>*Selection*: not stated.<br>*Recruitment*: not stated.<br>*Consent***:** not stated.	Survey study with 22 participants.	*Reliability*: the use of structured survey may increase the collected data reliability.<br>*Validity*: An interface was designed to test and validate the findings of the survey.	Not stated.	*Reliability*: not stated.<br>*Validity*: not stated.	Found that users desired the ability to annotate, retroactively repair, and compare their data; thus, developed an interface based on these findings.	A, B, D, F, G, J, K, L

Pickard [143]	Investigate consumer attitudes toward sharing health information, particularly for research purposes.	Crowdsourced health study cohort.	*Sampling source:* members of Genomera and Traitwise. *Selection*: participants enrolled voluntarily into the study. *Recruitment*: via Genomera. *Consent*: sought from participants via online form.	Survey study consisted of 27 questions and was performed via Genomera from July 30, 2012 to April 30, 2013, with 128 participants.	*Reliability*: the use of structured survey could increase the collected data reliability. *Validity*: the study has re-opened on Genomera to collect further information to validate the results.	Descriptive and correlation analysis were conducted.	*Reliability*: not stated. *Validity*: cohort-based correlations were not possible due to limitation in the sample size.	Found a strong willingness to share personal health information. Suggested a framework to increase health information sharing, which describes four elements: trust, motivation, community, and informed consent.	A, B, C, D, E, F, G, I, J, K, L
Rooksby [6]	Investigate approaches to personal tracking associated with health and wellbeing.	Qualitative exploratory method used to collect data about personal tracking.	*Sampling source:* local subjects (Scotland city) *Selection*: people that used, had used, or would like to borrow and use a pedometer or activity tracker were eligible to participate. *Recruitment*: via posters advertising the study in coffee shops, a bookshop, and a university building in a city in Scotland. *Consent*: not stated.	Interview study with 22 activity tracker users, and a follow-up contact with 22. Email and video conferencing were used to contact participants.	*Reliability*: the use of unstructured interviews may reduce the collected data reliability. *Validity*: mixed qualitative methods used to validate the results.	The interviews were transcribed and thematically analysed.	*Reliability*: Not stated. *Validity*: data analysis was grounded in the views of interviewees.	Suggested five styles of personal tracking: directive tracking, documentary tracking, diagnostic tracking, collecting rewards, and fetishised tracking.	A, B, C, D, E, F, G, H, I, J, K, L
Whooley [129]	Investigate how members of the QuantifiedSelf Meetup Group overcome data integration barriers.	Qualitative research was conducted by collecting data from presentations posted on Quantifies-self Meetup site.	*Sampling source:* members of Quantified Self Meetup group. *Selection*: video- recordings posted on Quantified-Self Meetup site. *Recruitment*: NA *Consent*: NA	51 videos of QuantifiedSelf presentations.	*Reliability*: systematic capturing of information about data integration. *Validity*: setting criteria of inclusion, and the observations were then reviewed, challenged, and refined in weekly meetings among the three authors.	The video-recordings were transcribed and thematically analysed.	*Reliability*: themes were analysed according to methods advocated by Neuman. *Validity*: use of Miles and Huberman framework to test the results of the qualitative analysis of video-recordings.	Identified three methods for data representation - binary, structured and abstract - that were shaped by users intentions of self-tracking and influenced reflection.	A, B, C, D, E, F, G, H, I, J, K, L
**2013**									
De Maeyer [7]	Investigate the awareness aspect of self-monitoring that the devices create in relation to the participants' ability to reach their goals.	Field study.	*Sampling source:* users of Body Media system who tracked physical activity, food and sleep. *Selection*: people who are not early adopters of self-monitoring devices were selected to participate in the study. *Recruitment*: via contacts of one of the authors.	Field study with10 participants wearing Body Media armbands and using the Body Media Activity manager to monitor their progress and goals for two periods (fall and spring) of	*Reliability*: systematic extraction of variables was conducted. *Validity*: setting criteria of inclusion increased the validity of data collection.	Observations were thematically analysed.	*Reliability*: data were analysed and results compared using Fogg's Behaviour Grid. *Validity*: results from period one were tested in period two.	Found that using self-tracking devices made participants more aware about their behaviour, and most of them also acted on this knowledge. The participants also improved their self-esteem because they achieved their initial goals.	A, B, C, D, E, F, G, H, I, J, K, L

			Consent: not stated.	two months.					
Fiore-Silfvast [10]	Examine gaps between discourses on data, practices with and around data, and contexts in which data exist; in order to define the nature of data in health self-tracking.	Exploratory method used to collect data about health monitoring.	*Sampling source:* various stakeholders associated with the use of technology in health and wellness.<br>*Selection*: formal healthcare institutions and informal consumer health communities were selected.<br>*Recruitment*: via Medicare project in Washington State, meetups groups like Quantified Self, and conferences such as Health 2.0, TedMed, and Medicine X.<br>*Consent*: not stated.	43 clinicians, technology designers, and users were interviewed.	*Reliability*: not stated.<br>*Validity*: not stated.	Not stated.	*Reliability*: not stated.<br>*Validity*: not stated.	Identified six data valences: self-evidence; actionability; communication/connection; transparency; truthiness; and discovery; that map the performance of self-tracking across the discourses, practices, and contexts of communities of practice.	A, B, C, D, J, K, L
Gimpe [13]	Investigate underlying motivations of self-triggered health monitoring.	Exploratory method used to collect data about health monitoring.	*Sampling source:* members of Quantified Self Berlin Meetup.<br>*Selection*: people who track themselves were eligible to participate.<br>*Recruitment*: respondents were recruited offline and online via multiple channels: in-person Meetups related to self-tracking, Meetup.com online groups related to self-tracking, Facebook groups related to self-tracking and health-related community groups in general, Twitter with tracking-related hashtags and accounts.<br>*Consent*: not stated.	150 self-trackers took part in one of six face-to-face group interviews conducted at a Meetup of the Quantified Self Berlin Meetup Group in September 2012.	*Reliability*: followed the methodology described by Hinkin in collecting data.<br>*Validity*: the sample size was big enough for data analysis, and setting criteria of inclusion increased the validity of data collection.	Factor analysis was conducted.	*Reliability*: followed the methodology described by Hinkin in analysing data to ensure reliability.<br>*Validity*: construct validity was assessed using Cronbach's alpha, and results were found to have adequate internal consistency and validity.	Developed a five-factor-framework of self-tracking motivations, and a psychometric scale of 19 items to measure individual drivers for self-tracking.	A, B, C, D, E, G, H, I, J, K, L
Guo [139]	Provide criteria to evaluate self-tracking devices available in the market, for researchers considering incorporating such tools into their research.	Field study was designed to evaluate tools' ability to measure number of steps and distance travelled by users.	*Sampling source:* participants who used one or more of these tools (i.e., Fitbit, Nike+ fuelband, Nike+ sportsband, Moves app, Omron steps pedometer, and SM-2000 pedometer).<br>*Selection*: participants were selected by the researcher.<br>*Recruitment*: via researcher's contacts.<br>*Consent*: not stated.	15 participants wore one or more of the selected set of devices. Some wore the tools for several weeks.	*Reliability*: systematic data collection was conducted.<br>*Validity*: objective data that were collected by SQ tools were used in the study.	Descriptive and correlation statistical analysis were conducted to analyse the datasets.	*Reliability*: the output of each device was instantly recorded after each use to ensure the reliability of the collected data.<br>*Validity*: normalised cross correlation of the data gathered was conducted, and only significant results were	Provide information about these devices for researchers considering incorporating these activity-based data streams into their research and for getting a better idea of the reliability and accuracy for use in life-logging and other multimedia applications.	A, B, C, D, E, F, G, H, I, J, K, L

							reported.		
Lee [9]	Investigate experiences of athletes already using physical activity tracking technologies.	Qualitative exploratory method used to collect data about personal tracking.	*Sampling source:* athletes.<br>*Selection*: participants who resided in and participated in athletic activities in Utah State were invited to participate.<br>*Recruitment*: via advertising on local sports equipment stores, running and cycling clubs, and word of mouth.<br>*Consent*: not stated.	Interview study with 20 athletes who participated in distance cycling or endurance running.	*Reliability*: structured interviews were conducted.<br>*Validity*: the sample size was big enough for data analysis, and setting criteria of inclusion increased the validity of data collection.	The interviews were transcribed and thematically analysed.	*Reliability*: three phases of thematic analysis were conducted iteratively to extract codes and themes.<br>*Validity*: two reports: numerical summaries of devices and data, and narrative descriptions of specific individuals were generated to test the extracted themes.	Found that data could affect athletes' performance, including creating tensions between their awareness of records of actual performance and their self-perception as athletic individuals.	A, B, C, D, E, G, H, I, J, K, L
Lee [141]	Investigate the use of physical activities tools to undertake data analysis activities.	Field study collecting data from fifth-grade students during recess time.	*Sampling source:* fifth-grade students.<br>*Selection*: 12 fifth-grade students from the same class were selected.<br>*Recruitment*: not stated.<br>*Consent*: not stated.	Two weeks field study was conducted to observe students' behaviour by using video and audio recordings.	*Reliability*: participants' activities were recorded by using the computer's internal webcam and microphone.<br>*Validity*: the observations from the first week's group informed and influenced the second week's implementation.	Statistical analysis of transcripts was conducted.	*Reliability*: transcript excerpts were provided to ensure reliability.<br>*Validity*: subsequent analysis of transcripts was conducted to test the observations.	Found that students were sensitive toward the two measures of centre (i.e., Mean and Median) which resulted in increasing the competition aspect during the recess class, and encourage them to collect and analyse data about their own recess activities.	A, B, C, D, E, F, G, I, J, K, L
**2012**									
Chang [136]	Provide a method for evidence-based practice through using self-tracking tools.	Case study.	*Sampling source:* one athlete.<br>*Selection*: a 13-year-old female who was an athlete of national level calibre and a patient with chronic illness (hypothyroidism).<br>*Recruitment*: the subject was chosen and contacted by the researcher.<br>*Consent***:** not stated.	Four experiments spanning seven years' worth of observations were conducted to test assumptions about exercise, diet, sleep, and medical treatment of hypothyroidism.	*Reliability*: data was collected systematically during the experiment.<br>*Validity*: the experiments were conducted by a single participant to ensure data consistency and validity of the collected data.	Descriptive and correlation statistical analysis were conducted to analyse the datasets.	*Reliability*: use of statistical software to analyse the datasets.<br>*Validity*: hypotheses were validated via correlation statistical analysis, and some were validated based on laboratory	Results indicate two areas in which pre-existing assumptions were not supported (diet and hypothyroidism), and two other areas in which further investigation was warranted (exercise and sleep).	A, B, C, D, E, F, G, H, I, J, K, L

							results.		
Li [4]	Investigate users experiences with collecting and reflecting on information obtained from personal informatics systems in order to provide guidance for making these systems more effective.	Exploratory qualitative research.	*Sampling source:* users of personal informatics systems. *Selection*: users who were more likely to have used one or more personal informatics systems. *Recruitment*: via blog dedicated to personal informatics (http://quantifiedself.com), a blog about general information visualisation (http://flowingdata.com), and forums at two personal informatics web sites (http://slifelabs.com and http://moodjam.org). *Consent*: not stated.	Online survey study with 68 participants, and interviews with 11 who agreed to participate in follow-up using Instant Messenger to collect additional details about their responses.	*Reliability*: use of structured survey to collect data. *Validity*: use of follow-up interviews to test and validate findings.	The survey and interviews were thematically analysed.	*Reliability*: systematic extraction of themes by using affinity diagrams from the transcripts. *Validity*: themes were identified from the data as the responses were processed.	Developed a stage-based model which represents a series of five stages, and barriers that people experienced in each stage.	A, B, C, D, E, F, G, H, I, J, K, L

* Key to quality criteria:

Quality of study reporting:
A: Aims and objectives clearly reported
B: Adequate description of context of research
C: Adequate description of the sample and sampling methods
D: Adequate description of data collection methods
E: Adequate description of data analysis methods

Trustworthiness, in other words, attempt to establish:
F: Reliability of data collection tools
G: Validity of data collection tools
H: Reliability of data analysis
I: Validity of data analysis

The appropriateness of the study design (i.e., in terms of the research aims of the present study):
J: Examined the use of SQ technologies for health self-management
K: Investigated users in real-world contexts or daily-life settings (e.g., not in controlled-environment laboratories) where people individually collected, managed, and/or reflected on data
L: Provided empirical evidence based on actual use (i.e. was not limited to proposing a new solution and reporting its technical specifications)

*NA Not Applicable
