# Supplementary material for: Activity Theory as a Theoretical Framework for Health Self-Quantification: A Systematic Review of Empirical Studies
Source: J Med Internet Res. 2016 May 27;18(5):e131. doi: 10.2196/jmir.5000 (PMC4909388; doi:10.2196/jmir.5000)
Supplement: Multimedia Appendix 2 [file jmir_v18i5e131_app2.pdf]

Year		Reasons for exclusion	
**First Author**	**Article reference number**	**Description of the technical aspect of the proposed application**	**General review of the field, commentary, or discussion of aspects of the Quantified Self movement**
**2015**			
Almalki	[1]		X
Andrieu	[20]		X
Banos	[21]		X
Barricelli	[22]	X	
Chamberlain	[23]		X
Davies	[24]		X
Doryab	[25]	X	
Dudley	[26]		X
Forsdyke	[27]		X
Giones-Valls	[28]		X
Hachem	[29]	X	
Hood	[30]		X
Issa	[31]		X
Janssen	[32]		X
Jones	[33]		X
Lavalliere	[34]		X
Lupton (a)	[35]		X
Lupton (b)	[36]		X
Majmudar	[37]		X
Ohlin	[38]		X
Olivier	[39]		X
Picard	[40]		X
Ruping	[41]		X
Thornquist	[42]		X
Tory	[43]		X
Van den Bulck	[44]		X
Williams	[45]		X
Zandi	[46]	X	
**2014**			
Almalki	[155]		X
Appelboom	[47]		X
Becker	[48]		X
Brennan	[49]	X	
Bushhousen	[50]		X
Caldwell	[51]		X
Calikli	[52]		X
Cena	[53]		X
Clifford	[54]		X
Cuttone (a)	[55]	X	
Cuttone (b)	[56]		X
De Croon	[57]		X
Duarte	[58]	X	
Gastaldi	[59]		X
Gaunt	[60]	X	
Gurrin	[61]		X
Harrison	[62]		X
Hirsch	[63]	X	
Hogenboom	[64]	X	
Huang	[65]	X	
Jain	[66]		X
Jones	[67]		X
Jordan	[68]		X
Keary	[69]		X
Khorakhun	[70]		X
Kido	[71]	X	
Lagus	[72]		X
Laundav	[73]	X	
Lee	[74]		X
Lingg	[75]	X	
Lupton (a)	[76]		X
Lupton (b)	[77]		X
Lupton (c)	[3]		X
Marcengo	[78]	X	
Maturo (a)	[79]		X
Maturo (b)	[80]		X
Meyer	[81]		X
Nafus	[82]		X
Nikolic-Popovic	[83]	X	
Prince	[84]		X
Rapp (a)	[85]		X
Rapp (b)	[86]		X
Reigeluth	[87]		X
Salamati	[88]		X
Saunders	[11]	X	
Schreier	[89]		X
Shull	[90]		X
Sjöklint	[91]		X

Wenger	[92]	X	
Zhu	[93]	X	
**2013**			
Altini	[94]	X	
Buzzo	[95]	X	
Calvo	[96]		X
Chen	[97]	X	
Cuttone	[98]	X	
Derksen	[99]	X	
Jain	[100]		X
Kido	[101]	X	
Kunze	[102]	X	
Lupton	[103]		X
Lupton	[104]		X
Martin-Sanchez	[105]		X
Matassa	[106]	X	
McFedries	[107]		X
Pasek	[108]		X
Salamati	[109]	X	
Swan	[110]		X
Yumak	[111]		X
**2012**			
Bottles	[112]		X
Lathia	[113]	X	
Lathia	[114]	X	
Li	[115]		X
Smarr	[116]		X
Rivera-Pelayo	[117]		X
Swan (a)	[118]		X
Swan (b)	[119]		X
Swan (c)	[120]		X
The Economist	[121]		X
**2009**			
Swan	[122]		X
